# Functional near-infrared spectroscopy can detect low-frequency hemodynamic oscillations in the prefrontal cortex during steady-state visual evoked potential-inducing periodic facial expression stimuli presentation

**DOI:** 10.1186/s42492-020-00065-7

**Published:** 2020-12-01

**Authors:** Meng-Yun Wang, Anzhe Yuan, Juan Zhang, Yutao Xiang, Zhen Yuan

**Affiliations:** 1 Faculty of Health Sciences, University of Macau, Taipa, Macau SAR, 999078 China; 2 Centre for Cognitive and Brain Sciences, University of Macau, Taipa, Macau SAR, 999078 China; 3 Eastside High School, 1201 SE 43rd Street, Gainesville, FL 32641 USA; 4 Faculty of Education, University of Macau, Taipa, Macau SAR, 999078 China

**Keywords:** Steady state visual evoked potentials, Dynamic facial expressions, Functional near-infrared spectroscopy, Brain oscillation

## Abstract

Brain oscillations are vital to cognitive functions, while disrupted oscillatory activity is linked to various brain disorders. Although high-frequency neural oscillations (> 1 Hz) have been extensively studied in cognition, the neural mechanisms underlying low-frequency hemodynamic oscillations (LFHO) < 1 Hz have not yet been fully explored. One way to examine oscillatory neural dynamics is to use a facial expression (FE) paradigm to induce steady-state visual evoked potentials (SSVEPs), which has been used in electroencephalography studies of high-frequency brain oscillation activity. In this study, LFHO during SSVEP-inducing periodic flickering stimuli presentation were inspected using functional near-infrared spectroscopy (fNIRS), in which hemodynamic responses in the prefrontal cortex were recorded while participants were passively viewing dynamic FEs flickering at 0.2 Hz. The fast Fourier analysis results demonstrated that the power exhibited monochronic peaks at 0.2 Hz across all channels, indicating that the periodic events successfully elicited LFHO in the prefrontal cortex. More importantly, measurement of LFHO can effectively distinguish the brain activation difference between different cognitive conditions, with happy FE presentation showing greater LFHO power than neutral FE presentation. These results demonstrate that stimuli flashing at a given frequency can induce LFHO in the prefrontal cortex, which provides new insights into the cognitive mechanisms involved in slow oscillation.

## Introduction

Brain oscillations are the instantiation of neuronal rhythms resulting from the dynamic reciprocity between intrinsic cellular and circuit properties [1], which can be ubiquitously detected across various spatial and temporal scales. Brain oscillations serve as a bridge linking single neuron activity to cognition and behaviors [1], in which the general functions of brain oscillators include input selection and plasticity, binding cell assemblies, consolidation, and a combination of learned information [1]. Disrupted brain oscillations, however, are considered a hallmark of numerous brain disorders, such as schizophrenia and autism [2]. Therefore, a thorough understanding of brain oscillations is essential to better decoding brain functions. The frequency band of brain oscillations is between 0.05 and 500 Hz [1]. In particular, high-frequency oscillations with frequencies over 1 Hz can be detected using electroencephalography (EEG) or magnetoencephalography, indicating that different canonical frequency bands such as delta (1-4 Hz), theta (4-8 Hz), alpha (8-12 Hz), beta (12-30 Hz), and gamma (> 30 Hz) correspond to distinct cognitive functions [2]. However, few studies have been carried out to inspect the cognitive functions associated with low-frequency oscillations, which generally fluctuate below 1 Hz. Meanwhile, low-frequency hemodynamic oscillations (LFHO) have been exploited using functional magnetic resonance imaging (fMRI) and functional near-infrared spectroscopy (fNIRS) [3–5], in which the low-frequency oscillation signals can be directly examined using the fast Fourier transform (FFT) of blood oxygenated (HbO) or deoxygenated (HbR) hemoglobin concentration change measurements. To date, it is still unclear whether neural activity is the major contributor to LFHO, since some studies also claim that LFHO in some frequency bands could be physiological noise [3]. One way to capture LFHO is to explore the role of brain oscillations in cognition [6]. For example, visual stimulus tasks can elicit steady-state visual evoked potentials (SSVEPs), which make them excellent candidates for inspecting high frequency oscillations [7] and LFHO [8].

More importantly, EEG and fMRI neuroimaging studies have been performed to inspect SSVEP in response to high-level cognitive processes, such as facial expression (FE) recognition [9–19]. Interestingly, previous fNIRS-based SSVEP studies mainly focused on the investigation of brain computer interfaces or brain activation in the occipital cortex [20, 21]. In contrast, the application of SSVEP in the fNIRS field [22–25] has never been used to explore how FEs relate to brain functions in the prefrontal cortex.

In this study, LFHO in the prefrontal cortex during SSVEP-inducing stimulation were inspected using fNIRS, in which the periodic flickering stimuli were FEs. In particular, FFT analysis and canonical hemodynamic response function (HRF) deconvolution were carried out to extract highly detailed spatial information on the power and phase of the HbO signals. It is hypothesized that LFHO can be induced by SSVEP-inducing flickering stimuli. It is also assumed that LFHO measures can be used to differentiate different FEs. It is expected that the present findings may contribute to a better characterization and understanding of the underlying neural mechanisms associated with LFHO.

## Methods

### Participants

Twenty-one college students (4 males and 17 females, mean age ± standard deviation: 22 ± 2.5 years) were recruited from the University of Macau campus. All participants were right-handed with normal vision. In addition, no history of neurologic or psychiatric disorders was reported for any participant. The present experimental tests were approved by the Biomedical Ethics Board of the University of Macau.

### Stimuli and procedures

**Fig. 1:**
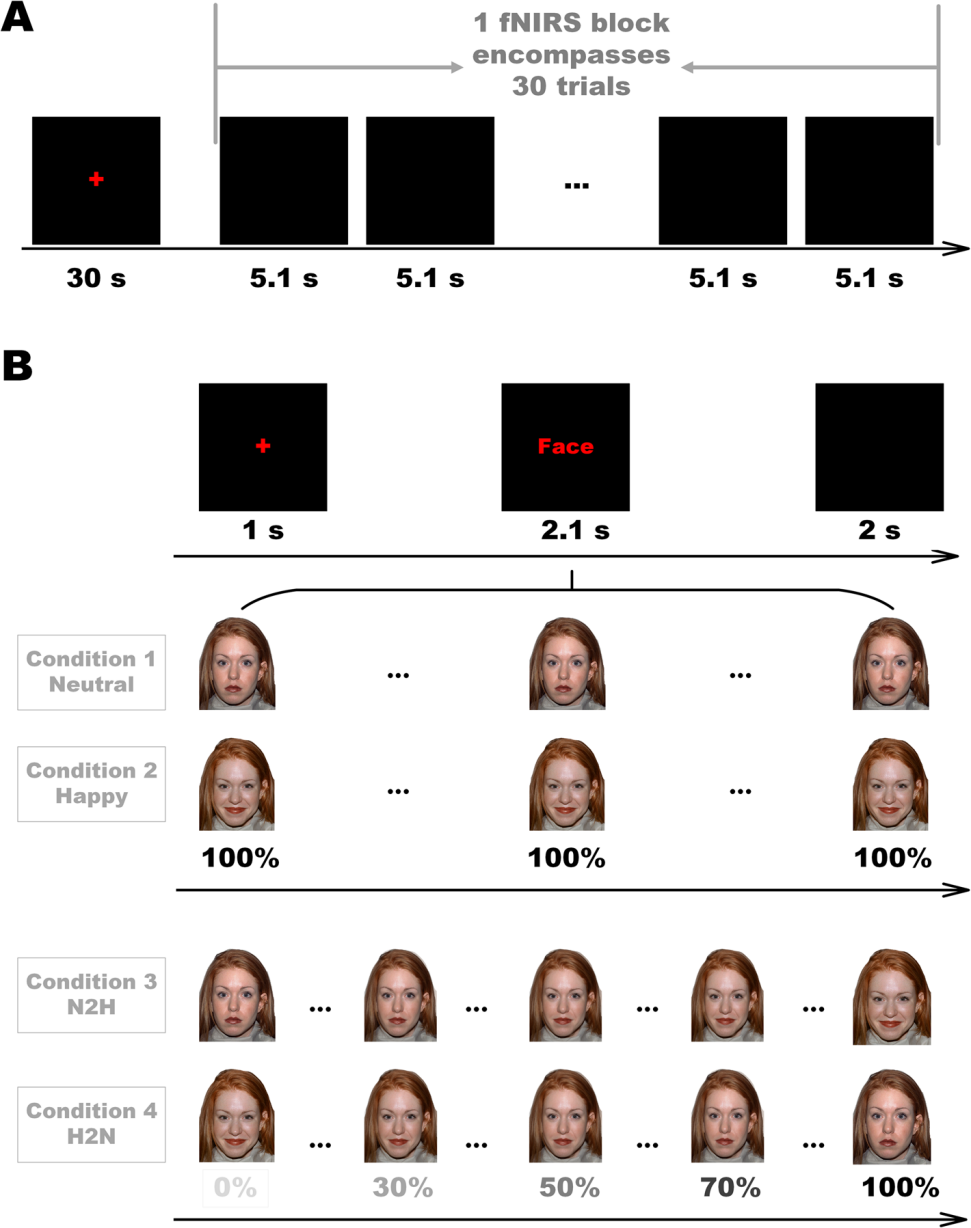
The procedure of the experiment. **a** The illustration of one fNIRS block including 30 trials; **b** The schema of one example trial

Two categories of facial stimuli (neutral and happy faces) were selected from the NimStim Face Stimulus Set [26]. Based on the original faces, morphed faces were produced by overlaying neutral faces with happy faces, leading to 21 FEs created with a 5% gradient change (Morpheus software). For example, the 30% happy to neutral (H2N) FE indicated that the stimulation material was generated with a 70% happy face and 30% neutral one (Fig. 1).

fNIRS data were recorded while the participants were performing the FE task. The fNIRS task in Fig. 1a consisted of four blocks (conditions): neutral (condition 1), happy (condition 2), neutral to happy (N2H; condition 3) in which a neutral face changed little by little to become a happy face, and H2N (condition 4), in which a happy face gradually became a neutral face. Each block had 30 trials and each trial lasted 5.1 s. The order of block presentations was completely random, and the rest period between each of the two blocks was 30 s. Therefore, the stimulus frequency of each block was set at 0.2 Hz. Each trial started with a red cross being displayed at the center of the monitor for 1000 ms, followed by 21 FE presentations for 2100 ms and a black screen for 2000 ms (Fig. 1b). In particular, the duration for each of the 21 FE presentation was 100 ms, which included a 30 ms presentation of an FE and 70 ms presentation of a black screen to generate the flashing effect. For each block, the FE presentations for each of the 30 trials were identical.

### fNIRS data acquisition and preprocessing

**Fig. 2:**
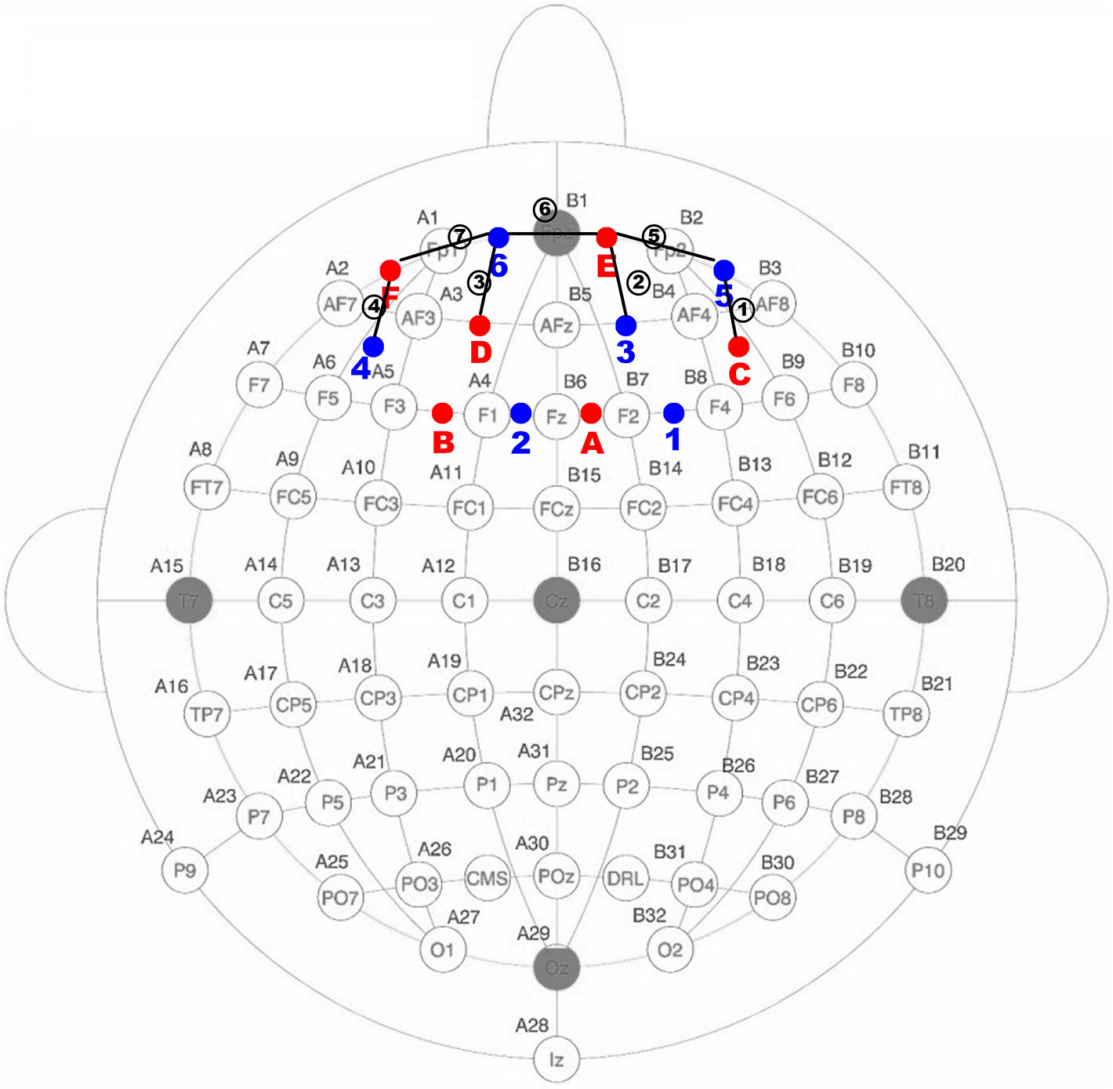
Configuration of fNIRS probes. Red and blue dots denote the laser sources and detectors, respectively. The connection between each laser source and detector is the channel

The fNIRS data were acquired at a sampling rate of 50 Hz using our CW6 system (Techen Inc., Milford, MA) [27–30]. Four laser sources at wavelengths of 690 nm and 830 nm and four light detectors were placed on a standard EEG cap to generate seven channels (Fig. 2), in which the spatial distance between each laser source and each detector was 3 cm. It should be noted that the fNIRS channels were located in the exact positions of the EEG electrodes: C-5 (Ch 01), E-3 (Ch 02), D-6 (Ch 03), F-4 (Ch 04), E-5 (Ch 05), E-6 (Ch 06), and F-6 (Ch 07).

**Fig. 3:**
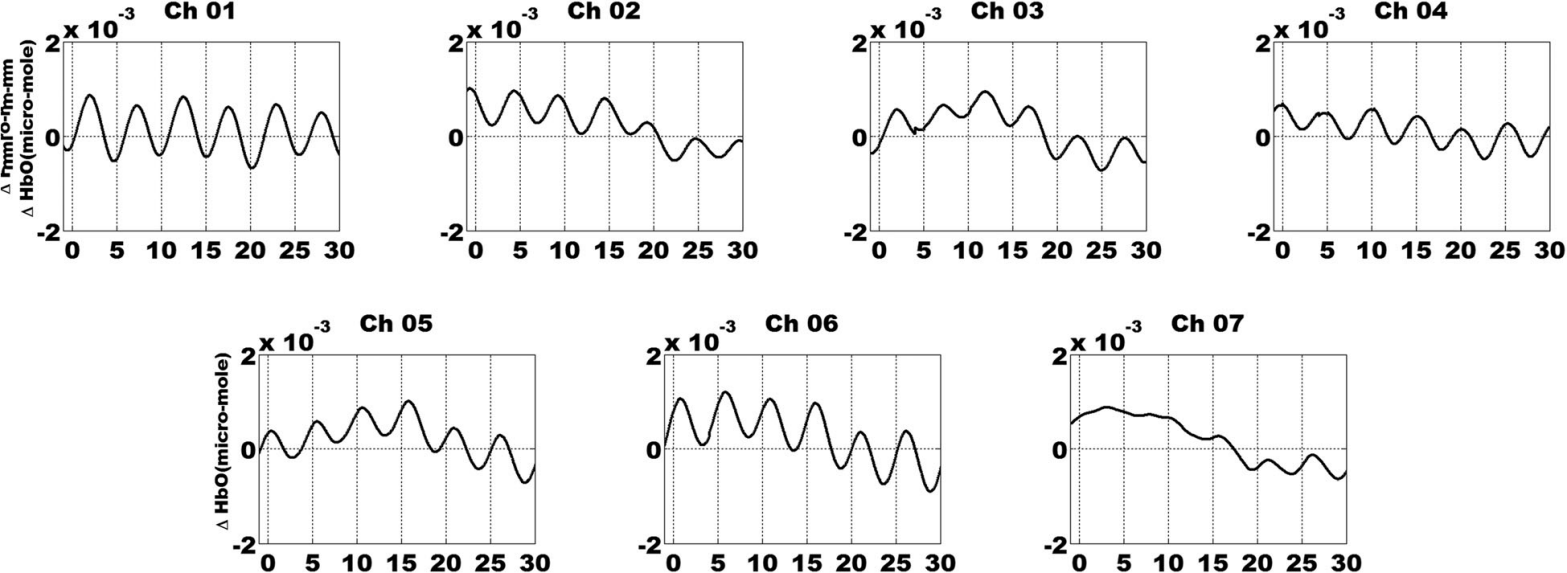
The time-domain of HbO signals during periodic SSVEP-inducing FE stimuli presentation. The time course of fNIRS data is grand-averaged across all conditions and participants. The unit of the *x*-axis is seconds while the unit of the *y*-axis is micromoles

The raw fNIRS data were first transformed to light density changes measured as optical density (OD) according to the modified Beer-Lambert law and then motion-corrected [31]. Further, a low bandpass filter at 0.25 Hz and then a high bandpass filter at 0.02 Hz were used to eliminate the effect of physiological noise. The high-pass filter removes low-frequency measurement noise while the low-pass filters remove physiological noise, such as heartbeat (1 Hz) and respiratory signals (0.25-0.3 Hz). Subsequently, the data were segmented into 5.1 s epochs for each trial consisting of 1 s before and 4.1 s after trigger onset. Finally, HbO and HbR hemoglobin changes were generated using OD values, and only HbO signals were analyzed in this study because they can serve as more sensitive indicators of changes associated with regional cerebral blood flow. The grand-averaged HbO signals across all conditions and participants are provided in Fig. 3 for the seven channels.

### Data analysis

In this study, the group-averaged HbO time series from seven channels were converted to frequency-domain signals via FFT. The FFT is usually applied to generate SSVEPs in EEG analysis, and was also used here to calculate the distributions of LFHO in the frequency domain. In addition, the signal noise ratio (SNR) of LFHO was computed here, which was equal to the power of a specific frequency divided by the averaged power of the 20 surrounding frequency bins taking the 10 nearest ones on each side [16, 32]. Further, the grand-averaged SNR time series of each channel was calculated for each condition across all participants. Importantly, the z-score of the grand-averaged SNR was generated for each condition, which was denoted as the power of a frequency that was first subtracted from the mean of 20 surrounding frequency bins and then divided by its standard deviation [16, 32].

## Results

### LFHO across the prefrontal cortex

**Fig. 4:**
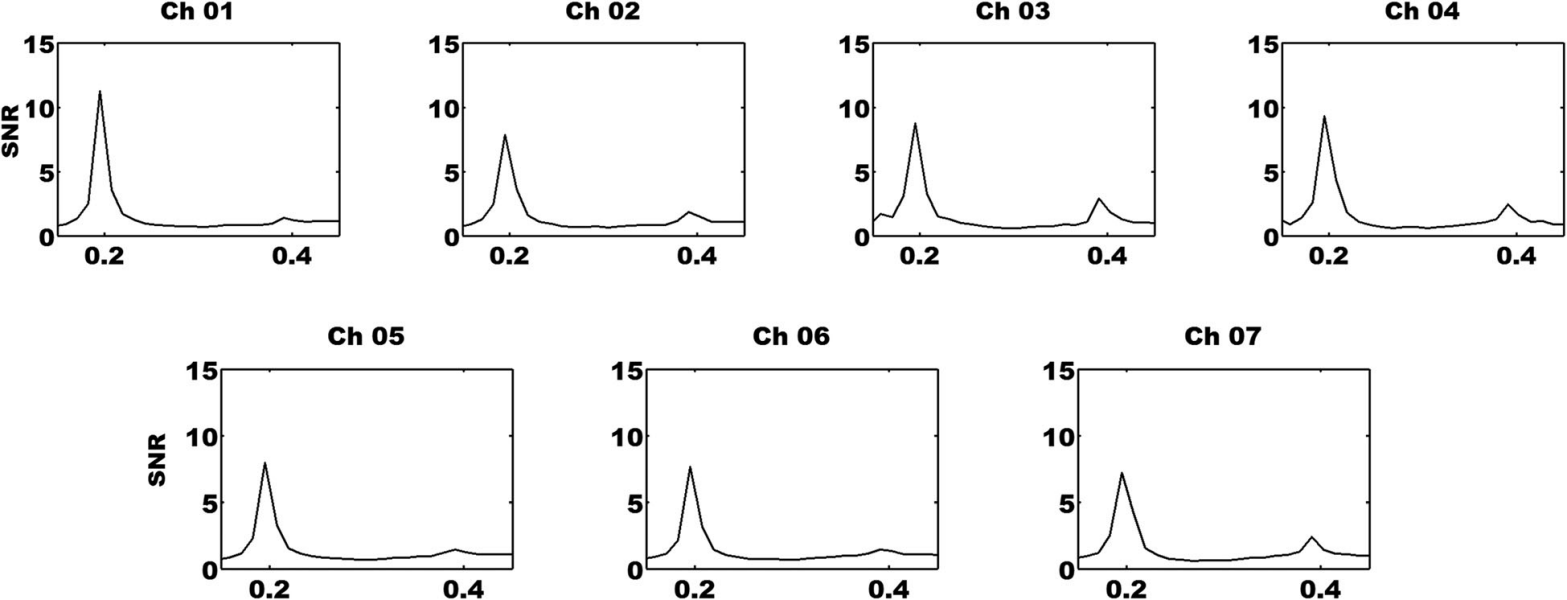
The frequency domain grand-averaged LFHO signals across all conditions and participants during periodic FE stimuli with SSVEP. The unit of *x*-axis is Hz in frequency while the *y*-axis has no units

As depicted in Fig. 4, LFHO were successfully evoked with periodic stimuli presentation at a frequency of 0.2 Hz for all channels. The LFHO exhibited a very similar distribution to the SSVEP, in which grand-averaged SNR peaks were detected at the fundamental frequency of the stimuli (0.2 Hz) and its harmonics (0.4 Hz).

**Fig. 5:**
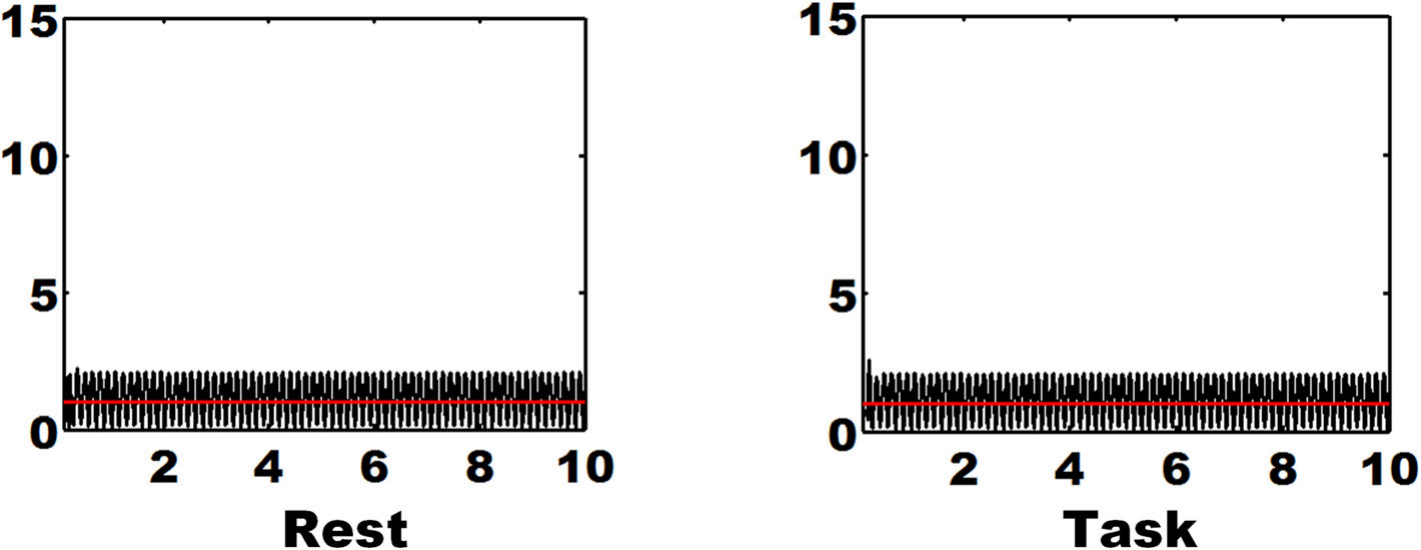
The general frequency-domain resting-state (left) or task-elicited (right) fNIRS data without periodic FE stimuli with SSVEP. The unit of the *x*-axis is seconds while the *y*-axis has no units

In addition, resting-state and task-elicited fNIRS recordings without flickering stimuli with SSVEP were also acquired in the frontal area using our CW6 system [24, 25]. The two sets of fNIRS data were also preprocessed and analyzed using the same procedure as LFHO during SSVEP-inducing periodic FE stimuli presentation. As displayed in Fig. 5, no monochronic peaks in SNR at a frequency of 0.2 Hz were identified for either fNIRS dataset. It was discovered that without external periodic stimuli, no LFHO at the dominant frequency can be elicited in the prefrontal cortex.

### LFHO after deconvolution

**Fig. 6:**
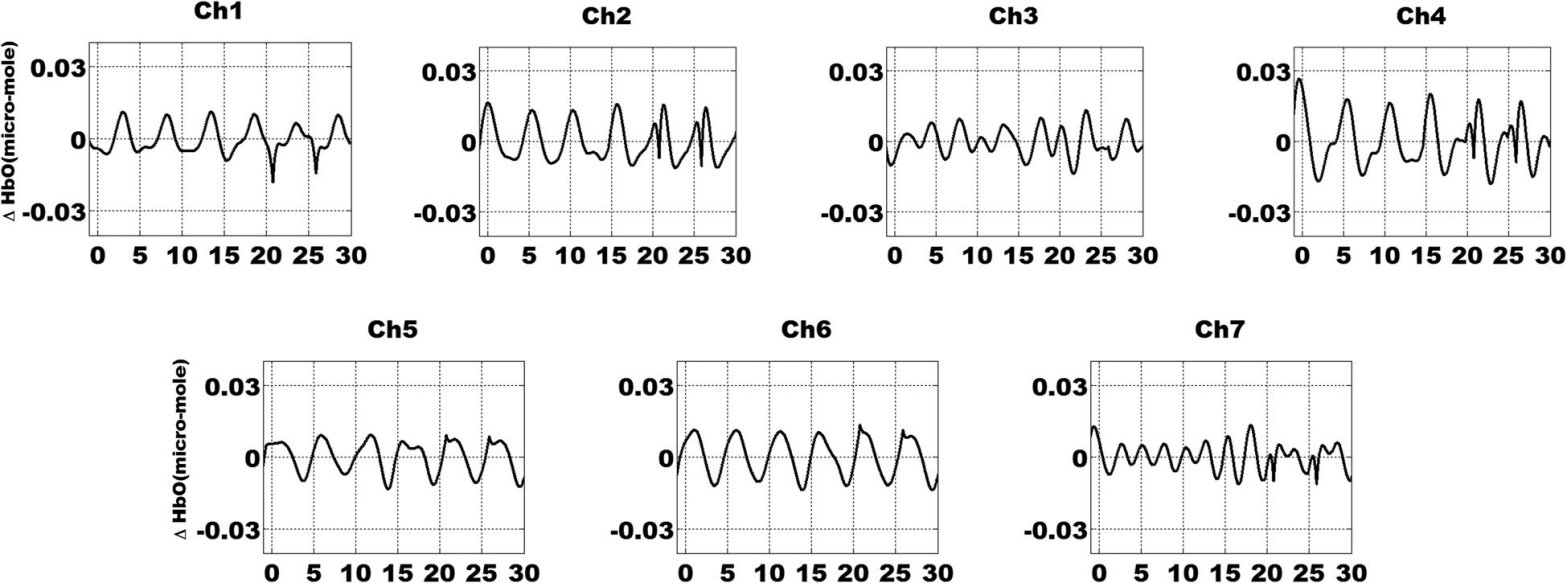
The time-domain of HbO signals after deconvolution during periodic FE stimuli with SSVEP. The time course of fNIRS data is grand-averaged across all conditions and participants. The unit of the *x*-axis is seconds while the unit of the *y*-axis is micromoles

**Fig. 7:**
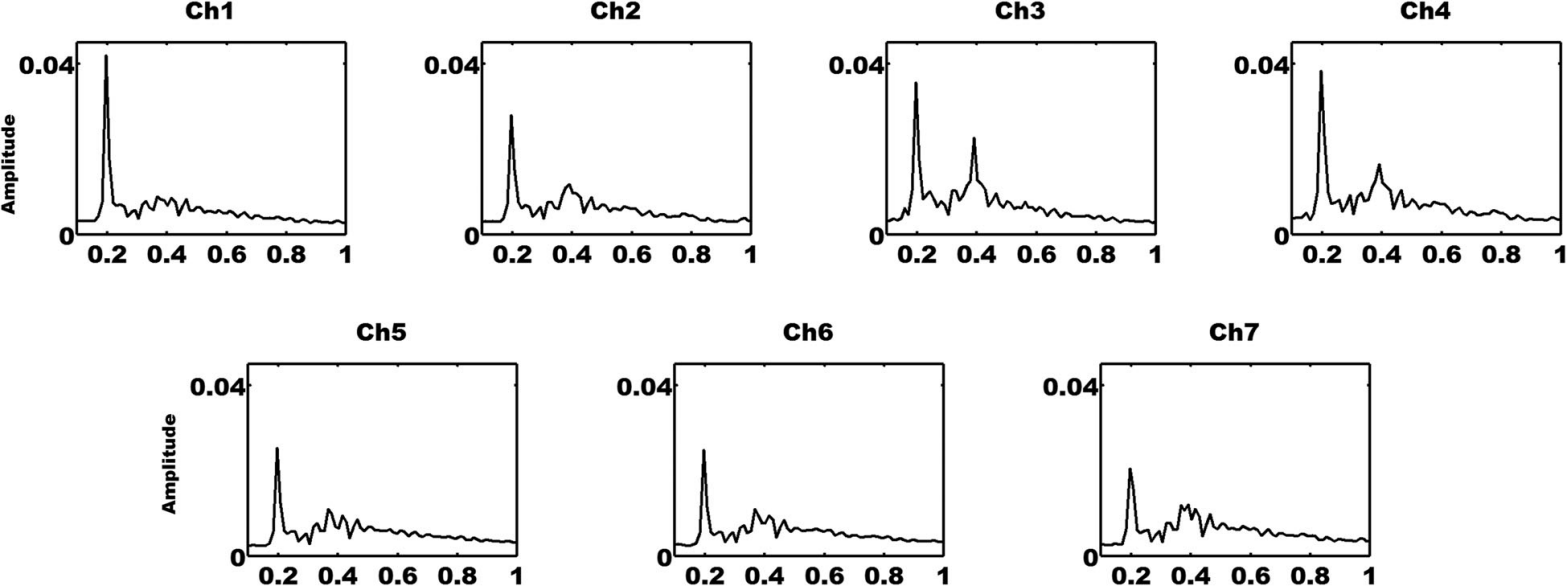
The frequency domain grand-averaged LFHO signals after deconvolution across all conditions and participants during SSVEP-inducing periodic FE stimuli presentation. The unit of the *x*-axis is Hz while the *y*-axis has no units

In addition, to inspect whether LFHO were independent of neurovascular coupling, HRF deconvolution was carried out to generate the “neural level signals” (LFHO after HRF deconvolution). The HRF was produced for each channel by matching the HbO with the canonical HRF and its time derivative. The blind HRF deconvolution is capable of eliminating the effect of hemodynamic responses to a maximal extent, in which the HRF is based on the convolution of the boxcar function and the sum of two gamma functions as the canonical HRF [4, 33]. The deconvoluted HbO data before and after FFT are plotted in Figs. 6 and 7, respectively. It was discovered that LFHO were detected at 0.2 Hz. The HbO/SNR signals from all channels before and after HRF deconvolution were compared under various conditions. It was discovered that the distributions of the SNR did not exhibit significant differences after HRF deconvolution, which demonstrates that LFHO are independent of neurovascular coupling.

### FE results

**Fig. 8:**
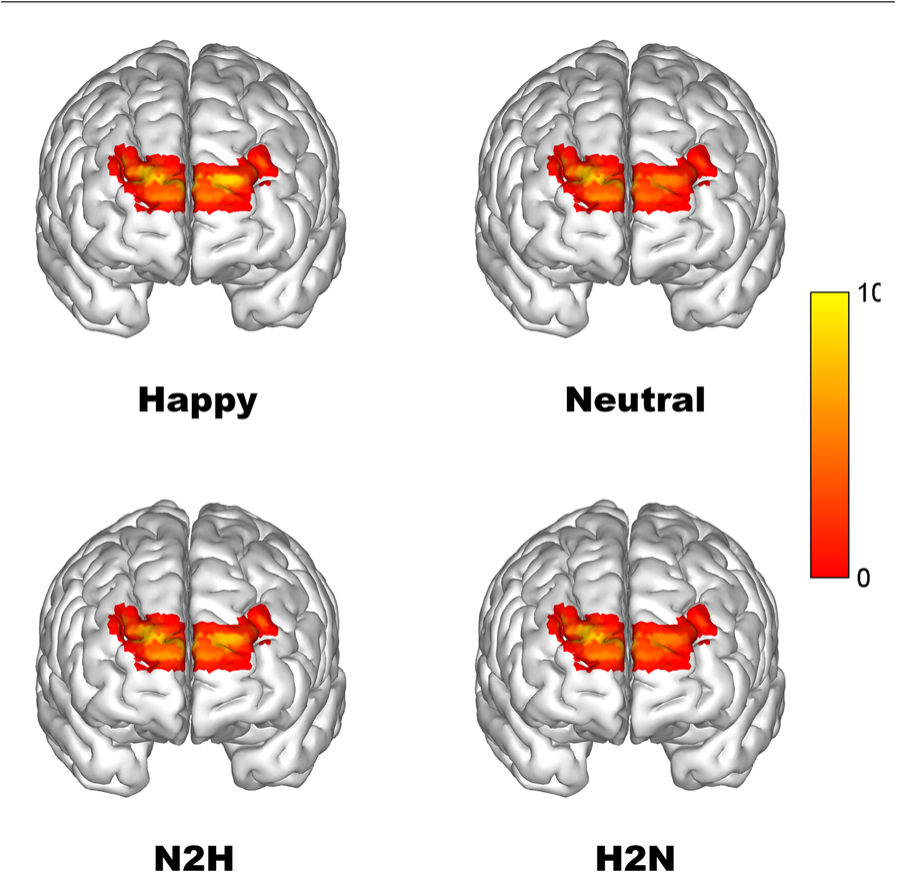
The brain activation maps of various conditions at a frequency of 0.2 Hz. The values are z-scores

Meanwhile, paired *t*-tests were performed to examine the difference in SNR amplitude of the dominant frequency (0.2 Hz for the present study) between the neutral and happy conditions, as well as the difference between the N2H and H2N conditions. All statistical analyses were conducted using SPSS 18.0 software. The SNR maps across all channels under different conditions are depicted in Fig. 8. Paired *t*-tests (*t*_15_ = 2.20, *p* = 0.044, CI = 0.09-5.54) demonstrated that the happy condition (13.73 ± 5.38) manifested greater brain activation than the neural condition (10.87 ± 5.21). By contrast, no significant difference between the N2H and H2N conditions was found (*t*_15_ = 0.36, *p* = 0.73, CI = − 4.15-5.83).

## Discussion

The main purpose of this study was to explore whether fNIRS can detect low-frequency oscillations elicited by periodic events in the prefrontal cortex. Accordingly, exogenous flashing FEs at 0.2 Hz were utilized as periodic stimuli to induce SSVEP. The FFT results of the HbO data show that there were predominant peaks of power at 0.2 Hz across all channels in the prefrontal cortex, indicating that LFHO were successfully evoked as a new indicator of cognitive function or brain disorders. More importantly, the power amplitudes at 0.2 Hz can exhibit differences in brain activation between different cognitive states; the happy condition elicited larger power amplitudes than the neutral condition. Interestingly, previous EEG studies also demonstrated that emotional stimuli elicited larger SSVEPs than neutral stimuli [11–15, 19].

It is widely recognized that SSVEPs are elicited mainly in electrodes over the occipital and parietal cortex, which exhibit oscillatory components when the participant is watching flickering at a constant frequency. However, previous studies mainly focused on the investigation of high-frequency neural oscillation activity with a frequency above 1 Hz. In contrast, low-frequency oscillations (lower than 1 Hz) are seldom examined given that EEG is not an ideal tool to inspect low-frequency oscillations. This study demonstrated that fNIRS is an ideal tool to evaluate LFHO in the prefrontal cortex that are related to high-level cognition, such as processing emotional FEs. Of note, due to the relatively low spatial resolution of fNIRS, the accuracy could be improved by the simultaneous utilization of Fourier and image space analysis [34, 35].

## Conclusions

Although fNIRS can only indirectly record neural activity, it was discovered that LFHO detected by fNIRS somehow reflect the underlying neural activity and are independent of neurovascular coupling. Given that the peak of LFHO at 0.2 Hz was induced by periodic FEs flickering at the same frequency, we infer that the high power amplitude of the happy condition might result from the underlying neutral activity rather than other physiological noise.

## Data Availability

The datasets used and/or analysed during the current study are available from the corresponding author on reasonable request.

## Abbreviations

EEG: Electroencephalography
FE: Facial expression
FFT: Fast Fourier transform
fMRI: Functional magnetic resonance imaging
fNIRS: Functional near-infrared spectroscopy
H2N: Happy to neutral
HbO: Oxygenated hemoglobin
HbR: Deoxygenated hemoglobin
HRF: Hemodynamic response function
LFHO: Low-frequency hemodynamic oscillations
N2H: Neutral to happy
OD: Optical density
SNR: Signal noise ratio
SSVEP: Steady state visual evoked potentials
